# Sex-specific associations between nine metal mixtures in urine and urine flow rate in US adults: NHANES 2009–2018

**DOI:** 10.3389/fpubh.2023.1241971

**Published:** 2023-07-28

**Authors:** Shuai Zhang, Hanhan Tang, Minglian Zhou

**Affiliations:** ^1^Department of Male Reproductive Health, Lianyungang Maternal and Child Health Hospital, Lianyungang, China; ^2^Clinical Center of Reproductive Medicine, Lianyungang Maternal and Child Health Hospital, Lianyungang, China; ^3^Graduate School of Xuzhou Medical University, Xuzhou Medical University, Xuzhou, China

**Keywords:** urine flow rate, metal mixtures, BKMR, WQS, qgcomp, NHANES, US adults

## Abstract

**Background:**

The urinary system serves as a crucial pathway for eliminating metallic substances from the body, making it susceptible to the effects of metal exposure. However, limited research has explored the association between metal mixtures and bladder function. This study aims to investigate the relationship between urinary metal mixtures (specifically barium, cadmium, cobalt, cesium, molybdenum, lead, antimony, thallium, and tungsten) and urine flow rate (UFR) in the general population, utilizing multiple mixture analysis models.

**Methods:**

This study utilizes data obtained from the National Health and Nutrition Examination Survey. After adjusting for relevant covariates, we assessed the correlations between metal mixtures and UFR using three distinct analysis models: weighted quantile sum (WQS), quantile g-computation (qgcomp), and Bayesian kernel machine regression (BKMR). Additionally, a gender-stratified analysis was conducted. Finally, we also performed sensitivity analyses.

**Results:**

A total of 7,733 subjects were included in this study, with 49% being male. The WQS regression model, when fitted in the positive direction, did not yield any significant correlations in the overall population or in the male and female subgroups. However, when analyzed in the negative direction, the WQS index exhibited a negative correlation with UFR in the overall group (β = −0.078; 95% CI: −0.111, −0.045). Additionally, a significant negative correlation between the WQS index and UFR was observed in the female group (β = −0.108; 95% CI: −0.158, −0.059), while no significant correlation was found in the male group. The results obtained from the qgcomp regression model were consistent with those of the WQS regression model. Similarly, the BKMR regression model revealed a significant negative correlation trend between metal mixtures and UFR, with cadmium and antimony potentially playing key roles.

**Conclusion:**

Our study revealed a significant negative correlation between urinary metal mixture exposure and mean UFR in US adults, with notable gender differences. Specifically, higher urinary levels of cadmium and antimony were identified as potential key factors contributing to the decrease in mean UFR. These findings significantly contribute to the existing knowledge on the impact of metal mixtures on bladder function and provide valuable insights for safeguarding bladder health and preventing impaired bladder function.

## 1. Introduction

Heavy metals pose significant environmental and occupational hazards, being widely prevalent in the environment and capable of entering the human body through various routes and forms (1, 2). These non-essential metal elements are often resistant to degradation, and some of them can undergo redox reactions, leading to the formation of biologically active substances that exhibit metallotoxicity even at low doses. Consequently, they are implicated in the pathogenesis of various diseases, including cancer, cardiovascular diseases, neurological disorders (such as Alzheimer’s and Parkinson’s diseases), and chronic inflammatory diseases (3).

Urine production and excretion play a crucial role in human metabolism, serving as a primary route for eliminating most metals from the body. Consequently, the urinary system is inevitably influenced by the presence of metal metabolites. Previous research has demonstrated the nephrotoxic effects of several metal elements, including barium, chromium, cadmium, cobalt, copper, lead, mercury, platinum, and uranium (4–6). These metals can induce cellular oxidative stress, resulting in cell swelling and apoptosis (7, 8). However, studies investigating the impact of metallic elements in urine on human bladder function, particularly at low exposure levels, are limited and primarily confined to animal experimentation (9, 10). Additionally, gender-based disparities in the effects of metal exposure have been observed in some studies (11, 12). Nevertheless, it remains unknown whether such differences extend to bladder function. Furthermore, the analysis of metal mixtures in urine and their influence on bladder function is understudied, hindered by methodological limitations.

Urine biomonitoring is a preferred method for assessing chemical elements, metabolite exposures, and nutritional status due to its non-invasive nature and compatibility with modern analytical techniques. It is equally valuable for detecting metal elements. However, urine analyte concentrations are influenced by various factors beyond exposure, including sampling time, variations in toxicant metabolism kinetics, and physiological characteristics such as dilution changes (13). Therefore, when utilizing field urine samples for research purposes, additional data on urine flow rate (UFR) should be collected to ensure accurate interpretation of urine data (14). Since 2009, the National Health and Nutrition Examination Survey (NHANES) has been assessing the mean UFR of participants aged 6 years and older. The UFR is mainly regulated by the strength of contraction of the detrusor muscle and bladder outlet resistance (15–20), indirectly reflecting bladder function and state.

The objective of our study was to investigate the association between low-dose metal mixtures, specifically barium (Ba), cadmium (Cd), cobalt (Co), cesium (Cs), molybdenum (Mo), lead (Pb), antimony (Sb), thallium (Tl), and tungsten (W), and UFR in the general adult population. We conducted this analysis utilizing the NHANES dataset from 2009 to 2018, aiming to identify the metal elements within the mixture that may have the greatest impact on UFR.

## 2. Materials and methods

### 2.1. Design and participants

NHANES, organized by the National Center for Health Statistics (NCHS), is a cross-sectional survey research program aimed at evaluating the health and nutritional status of individuals in the United States, including both adults and children. Since 1999, NHANES has been conducted biennially using a complex multistage probability sampling design. The survey results are instrumental in determining the prevalence of major diseases and identifying associated risk factors (21). For this study, we utilized NHANES data from five cycles spanning 2009–2010, 2011–2012, 2013–2014, 2015–2016, and 2017–2018. The NHANES study was conducted under the authorization of the National Center for Health Statistics (NCHS) Ethics Review Committee, and all participants provided informed consent.

All data utilized in this study are publicly available on the official NHANES website: https://www.cdc.gov/nchs/nhanes/index.htm (Last accessed on March 20, 2023). The initial enrollment consisted of 49,693 participants across the five cycles. After screening the data, individuals under the age of 20 were excluded (*N* = 20,858). Furthermore, participants with missing key data were excluded (missing urine metal data: *N* = 19,802; missing UFR data: *N* = 490), along with those with missing covariate data (*N* = 810). Ultimately, a total of 7,733 adult participants were included in this study.

### 2.2. Measurement of urine metals

Urine specimens were collected within mobile exam centers (MEC) and subsequently processed, stored, and transported to the Laboratory Sciences Department of the National Center for Environmental Health for analysis. The levels of metals in urine samples were directly measured using inductively coupled plasma mass spectrometry (ICP-MS), with a comprehensive description of the laboratory method provided in the NHANES official instruction document (22). To address values falling below the lower limit of detection (LLOD), the NHANES guidelines recommended replacing them with the LLOD divided by the arithmetic square root of 2 (22). The detection rates for all metal elements exceeded 75%, and a detailed breakdown of the detection rates for each specific metal element can be found in Supplementary Table S1.

### 2.3. Measurement of UFR

NHANES initiated the collection of UFR data in 2009. Participants were instructed to note the time of their last urination prior to visiting the MEC. Within the MEC, participants provided urine samples and documented the collection time and volume for UFR calculation. The composite urine sample’s UFR was determined using the equation: UFR = (total urine volume)/(total duration) (23). To ensure an adequate urine volume for various analyses, each participant was permitted to provide up to three urine samples. Comprehensive guidelines for urine collection and handling can be found in the NHANES Laboratory Procedures Manual (LPM).

### 2.4. Assessment of covariates

To control for the effect of confounding factors on the study results, covariate adjustment was performed in the data analysis. The following covariates were included: sex, age (continuous), race, educational attainment, BMI (categorical), smoking status (categorical), cardiac history (categorical), systolic blood pressure (continuous), urinary creatinine (continuous), serum glucose (continuous), aspartate aminotransferase (AST, continuous), and estimated glomerular filtration rate (eGFR, continuous). Race categories were Mexican American, non-Hispanic White, non-Hispanic Black, other Hispanic, and other races. Educational attainment categories were less than 9th grade, 9–11th grade, high school graduate/GED or equivalent, some college or AA degree, and college graduate or above. BMI categories were underweight (<18.5 kg/m2), normal (18.5 to <25 kg/m2), overweight (25 to <30 kg/m2), and obese (30 kg/m2 or greater). Smoking status was defined as never smoker, former smoker, and current smoker based on self-reported information. A history of cardiac disease was defined as a history of one or more of congestive heart failure, coronary artery disease, angina pectoris, and heart attack. Serum glucose levels were measured using the DxC800 modular chemistry system with a Beckman Oxygen electrode, while AST levels were measured using the DxC800 enzymatic rate method. eGFR was calculated using the modified 4-variable Modification of Diet in Renal Disease (MDRD) formula: eGFR (mL/min/m2) = 175 × (Scr)^−1.154^ × (age)^−0.203^ × 0.742 (if female) × 1.212 (if black), where Scr is the serum creatinine level (mg/dL) and age is expressed in years (24). Creatinine levels were measured using the Roche/Hitachi Modular P Chemistry Analyzer from serum and urine samples.

### 2.5. Statistical analysis

Since the elemental metal, UFR, and urinary creatinine data were seriously right-skewed, a natural logarithm (ln) transformation was applied to these variables in order to improve their distribution characteristics and minimize the effect of outliers (25). Quantitative data are presented as the Median (interquartile range, IQR), while qualitative data are reported as percentages. Spearman’s correlation test was employed to examine the associations between ln-transformed metals.

To assess the relationship between metal mixtures and UFR, we employed three advanced mixture analysis methods: Weighted quantile sum (WQS), Quantile g-computation (qgcomp), and Bayesian kernel machine regression (BKMR).

#### 2.5.1. WQS model

We used a WQS regression model (26, 27) to assess the effect of metal mixtures. This method realizes dimension reduction and solves the collinearity problem by constructing the WQS index, and further tests the association between the WQS index and outcome. The model assigned weights to each exposure variable to determine their relative importance in influencing the outcome and identify potential high-risk factors. The WQS regression assumes by default that all exposed variables are correlated with the outcome in the same direction (positive or negative). Therefore, in the actual number analysis, two runs are required to test for positive and negative correlations. During the model fitting process, the dataset was divided into a 40% training set and a 60% validation set. The training set was utilized for weight estimation, while the validation set was used to test the significance of the WQS index. The final WQS index of this study was averaged from the weights in the 500 bootstrap samples.

#### 2.5.2. Qgcomp model

The qgcomp model is a newly developed approach that integrates WQS regression with basic g calculation (28). By employing quantile g calculation, we can assess the overall effect on the results when all exposures are simultaneously increased by one quartile, irrespective of the direction of correlation between exposures and results. In cases where different metal elements exert distinct directional influences, qgcomp assigns positive or negative weight values to each metal element, which sum up to 1 or − 1.

#### 2.5.3. BKMR model

The BKMR model (29) was employed to investigate the potential nonlinear relationship between each metal element and UFR, as well as the combined impact of metal mixtures on UFR. This method has strong statistical power in the field of mixed contaminant analysis. By fixing all metals simultaneously at a specific percentile (ranging from the 25th to the 75th percentile) compared to when they are fixed at the median, we can obtain the overall effect of the metal mixture on UFR., we can obtain the overall effect of the metal mixture on UFR. By fixing other metal elements at their respective median levels, we examined the nonlinear correlation between exposure and outcome by looking at exposure-response cross-sections between specific metal elements and the outcome. When all other metals are fixed at the 25th percentile, 50th percentile, and 75th percentile, respectively, the individual effects of a single metal are shown by comparing the risk associated with the 75th percentile of a particular metal element to its 25th percentile. Additionally, the model calculates the posterior inclusion probability (PIP) for each metal. In this study, the model was run with 50,000 iterations of the Markov chain Monte Carlo sampler.

Given NHANES’ utilization of a complex multistage probability sampling design, we performed multiple linear regression analyses in a weighted setting to check the robustness of the results. We examined the relationship between urinary metallic elements and UFR using both monometallic and polymetallic models.

To assess potential gender differences in the relationship between metallic elements in urine and UFR, we performed a gender-stratified analysis that covered all models.

All the aforementioned analyses incorporated all covariates, including sex, age, race, educational attainment, BMI, smoking status, cardiac history, systolic blood pressure, ln-urine creatinine, serum glucose, AST, and eGFR. For the gender-stratified study, all covariates other than gender were included.

All statistical analyses were performed using R version 4.2.2. The weighted analysis utilized the “survey” package (version 4.1-1). The WQS regression model employed the “gWQS” package (version 3.0.4), the qgcomp model utilized the “qgcomp” package (version 2.10.1), and the BKMR model employed the “bkmr” package (version 0.2.2). For statistical significance, *p*-values (two-sided) below 0.05 were considered significant.

## 3. Results

### 3.1. Participant baseline characteristics

Table 1 presents the essential characteristics of the study population under investigation. The median age of the participants was 47.0 years. Among the included participants, 49% (*n* = 3,812) were male, with a median age of 46 years. Baseline comparisons revealed that male participants exhibited higher levels of systolic blood pressure, glucose, AST, urinary creatinine, and mean UFR. Additionally, more of the male participants had a cardiac history and smoking.

**Table 1:** Basic characteristics of the population included in this study (*N* = 7,733), NHANES, USA, 2009–2018.

	Overall	Sex group		
Characteristic	Overall, *N* = 7,733 (100%)^a^	Female, *N* = 3,921 (51%)^b^	Male, *N* = 3,812 (49%)^a^	*p*-value^b^
**Cycle (n%)**				>0.9
2009–2010	1,745 (19%)	886 (19%)	859 (19%)	
2011–2012	1,449 (20%)	714 (20%)	735 (19%)	
2013–2014	1,579 (20%)	808 (20%)	771 (20%)	
2015–2016	1,541 (20%)	787 (21%)	754 (20%)	
2017–2018	1,419 (21%)	726 (20%)	693 (21%)	
**Age (years)**	47.0 (33.0, 61.0)	48.0 (34.0, 61.0)	46.0 (33.0, 60.0)	0.024
**Sex [n (%)]**				
Female	3,921 (51%)			
Male	3,812 (49%)			
**Race [n (%)]**				0.074
Non-Hispanic White	3,100 (66%)	1,559 (66%)	1,541 (67%)	
Non-Hispanic Black	1,595 (11%)	781 (12%)	814 (9.8%)	
Mexican American	1,150 (8.6%)	598 (8.2%)	552 (9.1%)	
Other/multiracial	1,071 (8.3%)	539 (8.5%)	532 (8.1%)	
Other Hispanic	817 (6.1%)	444 (6.1%)	373 (6.1%)	
**BMI [n (%)]**				<0.001
Underweight (<18.5)	119 (1.5%)	75 (2.0%)	44 (0.9%)	
Normal (18.5 to <25)	2,100 (27%)	1,090 (31%)	1,010 (24%)	
Overweight (25 to <30)	2,521 (33%)	1,098 (28%)	1,423 (37%)	
Obese (30 or greater)	2,993 (39%)	1,658 (39%)	1,335 (38%)	
**Smoking status [n (%)]**				<0.001
Never smoker	4,355 (56%)	2,572 (63%)	1,783 (48%)	
Former smoker	1,884 (26%)	707 (20%)	1,177 (32%)	
Current smoker	1,494 (18%)	642 (17%)	852 (20%)	
**Education attainment [n (%)]**				0.034
Less than 9th grade	773 (5.2%)	380 (4.9%)	393 (5.6%)	
9–11th grade	998 (9.5%)	483 (9.1%)	515 (10.0%)	
High school grad/GED	1,756 (23%)	834 (22%)	922 (24%)	
Some college or AA degree	2,345 (32%)	1,298 (34%)	1,047 (30%)	
College graduate or above	1,861 (31%)	926 (31%)	935 (31%)	
**Cardiac history [n (%)]**				<0.001
Heart attack	588 (6.4%)	225 (5.1%)	363 (7.7%)	
Non heart attack	7,145 (94%)	3,696 (95%)	3,449 (92%)	
Systolic blood pressure (mmHg)	120 (111, 131)	117 (107, 131)	122 (114, 132)	<0.001
Serum glucose (mg/dL)	93 (85, 103)	92 (85, 101)	94 (86, 104)	<0.001
Urine creatinine (mg/dL)	99 (55, 158)	79 (43, 131)	123 (72, 179)	<0.001
Aspartate aminotransferase (U/L)	22 (19, 27)	21 (18, 25)	24 (20, 29)	<0.001
eGFR (mL/min/m2)	87.48 (74.00, 102.65)	87.42 (73.34, 103.73)	87.49 (74.87, 101.67)	>0.9
Urine flow rate (mL/min)	0.87 (0.55, 1.42)	0.83 (0.51, 1.43)	0.90 (0.59, 1.42)	0.001

a n (unweighted) (%); Median (IQR).

b Chi-squared test with Rao and Scott’s second-order correction; Wilcoxon rank-sum test for complex survey samples.

BMI, body mass index; eGFR, estimated glomerular filtration rate.

### 3.2. Metal correlation study

**Figure 1:**
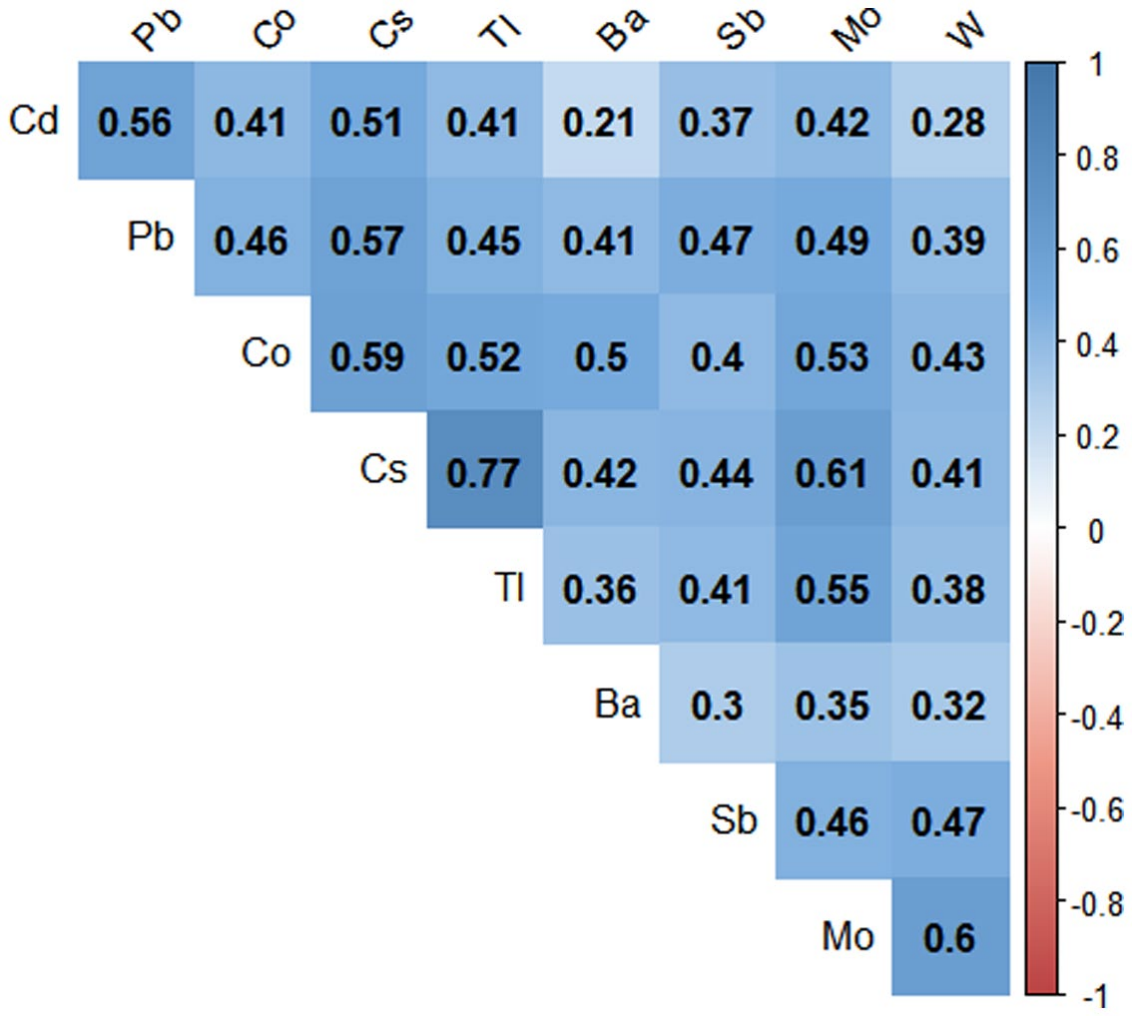
Paired Spearman correlations for urinary concentrations of nine metals in the population (*N* = 7,733), NHANES, USA, 2009–2018. The blue color represents the positive correlation, all correlations were statistically significant (*p* value < 0.001).

The Spearman correlation coefficients (rs) between the ln-transformed metals ranged from 0.21 to 0.77 (see Figure 1), with the strongest correlations being Cs with Tl (*r* = 0.77), and Cs with Mo (*r* = 0.61), Mo with W (*r* = 0.6), and Cs with Co (*r* = 0.59), respectively, with significant correlations for all metals (*p* < 0.001).

### 3.3. Differences in the distribution of metal elements in different groups

Supplementary Table S2 shows the distribution of metallic elements in urine in general and among different sexes. We revealed significant differences between males and females in the concentrations of several metal elements. Specifically, males exhibited notably higher levels of Ba, Cs, Mo, Pb, Sb, Tl, and W compared to females. Conversely, females displayed higher levels of Cd exposure.

### 3.4. WQS regression model and qgcomp model

**Figure 2:**
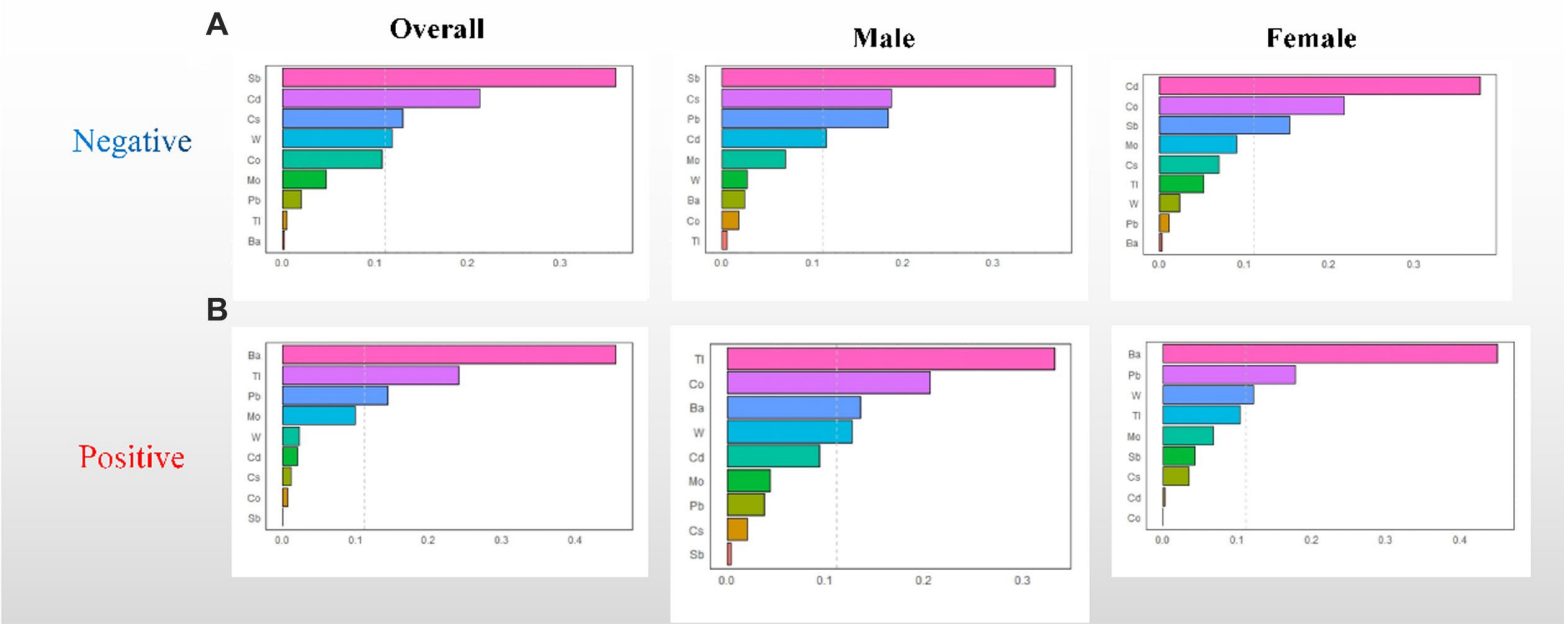
WQS regression weights of the urinary metals for UFR. **(A)** Negative WQS regression weights between urinary metals and UFR; **(B)** positive WQS regression weights between urinary metals and UFR. The model adjusted for sex, age, race, educational attainment, BMI, smoking status, cardiac history, systolic blood pressure, ln-urine creatinine, serum glucose, AST, and eGFR. Confounders other than sex were included in the gender-stratified analysis.

The WQS regression model was utilized to investigate the correlation between urine metal and UFR in both positive and negative directions. After adjusting for all confounding factors, no significant correlation was observed between the WQS index and UFR in the positive direction. However, in the negative direction, a significant negative correlation was found between the WQS index and UFR in the overall (β = −0.078; 95% CI: −0.111, −0.045). Subsequent gender-stratified analysis revealed a significant negative correlation between the WQS index and UFR in females (β = −0.108; 95% CI: −0.158, −0.059), while no significant correlation was observed in males (β = −0.014; 95% CI: −0.059, 0.032) (see Table 2). Additionally, Figure 2 shows the estimated weights for each WQS index, with Sb and Cd exhibiting the highest negative weights in the overall, and Cd, Co, and Sb showing the highest negative weights in females.

**Table 2:** Association between urine metal WQS index and qgcomp index and UFR (*N* = 7,733), NHANES, USA, 2009–2018.

	Negative WQS		Positive WQS		qgcomp	
	Beta (95%CI)	*p*-value	Beta (95%CI)	*p*-value	Beta (95%CI)	*p*-value
Overall	−0.078 (−0.111, −0.045)	<0.001	0.01 (−0.017, 0.036)	0.47	−0.061(−0.091, −0.031)	<0.001
Male	−0.014 (−0.059, 0.032)	0.563	−0.005 (−0.053, 0.042)	0.823	−0.011 (−0.054, 0.031)	0.601
Female	−0.108 (−0.158, −0.059)	<0.001	−0.023 (−0.061, 0.016)	0.245	−0.096 (−0.139, −0.053)	<0.001

Data are expressed as WQS regression indices (95% confidence intervals) and qgcomp model indices (95% confidence intervals). The model was adjusted for sex, age, race, educational attainment, BMI, smoking status, cardiac history, systolic blood pressure, ln-urine creatinine, serum glucose, AST, and eGFR. In analyses stratified by sex, the full model was adjusted for confounders other than sex. WQS, weighted quantile sum; qgcomp, quantile g-computation; CI, confidence interval.

**Figure 3:**
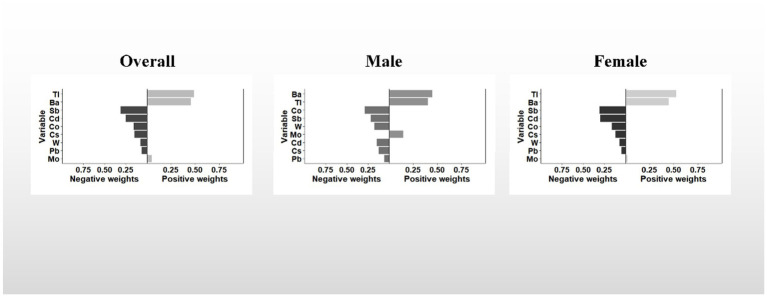
The qgcomp model weights of the urinary metal mixture for UFR. The model adjusted for sex, age, race, educational attainment, BMI, smoking status, cardiac history, systolic blood pressure, ln-urine creatinine, serum glucose, AST, and eGFR. Confounding factors other than sex were included in the gender-stratified analysis.

Similar to the results of the WQS model, the results of the qgcomp model showed a similar trend. In the overall, the qgcomp index exhibited a negative correlation with UFR (β = −0.061; 95% CI: −0.091, −0.031). Regarding single metal weights, urinary Tl (49%) had the highest positive contribution to the overall effect, followed by Ba (45.8%). Conversely, urinary Sb (30.8%) had the most negative weight, followed by Cd (25.1%). Similar to the findings from the WQS model, no significant association between the metal mixture and UFR was observed in males. However, in females, the qgcomp index showed a significant negative correlation with UFR (β = −0.096; 95% CI: −0.139, −0.053). In terms of single metal weights, urinary Tl (53%) made the largest positive contribution to the overall effect, followed by Ba (44.9%). Urinary Sb (30.8%) had the greatest negative weighting, followed by Cd (29.6%). For detailed results, refer to Table 2 and Figure 3.

### 3.5. BKMR model to assess the correlation between metal mixture and UFR

**Figure 4:**
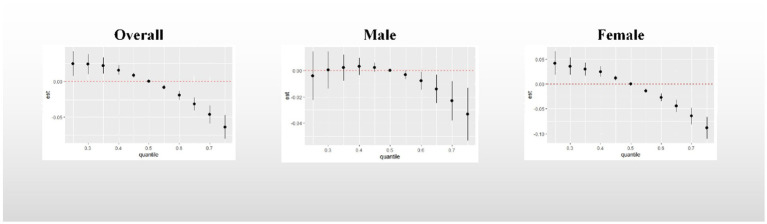
Overall association between metal mixtures and UFR. The model adjusted for sex, age, race, educational attainment, BMI, smoking status, cardiac history, systolic blood pressure, ln-urine creatinine, serum glucose, AST, and eGFR. Confounders other than sex were included in the gender-stratified analysis.

Overall association: Figure 4 demonstrates the overall association between the metal mixture and UFR. Upon adjusting for all confounding factors, a consistent decreasing trend was observed in the effect on UFR when the concentrations of all metals were simultaneously fixed at different percentiles (25th-75th percentiles) compared to when they were fixed at the median. Notably, the effect of the urinary metal mixture on UFR reached statistical significance only when all metals were simultaneously fixed at the 60th percentile and above among male participants. This finding indicates a significant negative correlation between urinary metal mixtures and UFR.

Nonlinear exposure-response relationships for single metals: Supplementary Figure S1 presents the nonlinear exposure-response relationships between single metals and UFR, with the concentrations of other metals held constant at their respective median concentrations. Ba exhibited a positive correlation with UFR in both the overall participants and among males. Conversely, Tl showed a positive correlation with UFR in females. Cd, Co, Cs, Sb, and W displayed negative correlations with UFR across all participants. Additionally, Pb demonstrated a negative correlation with UFR specifically among males.

**Figure 5:**
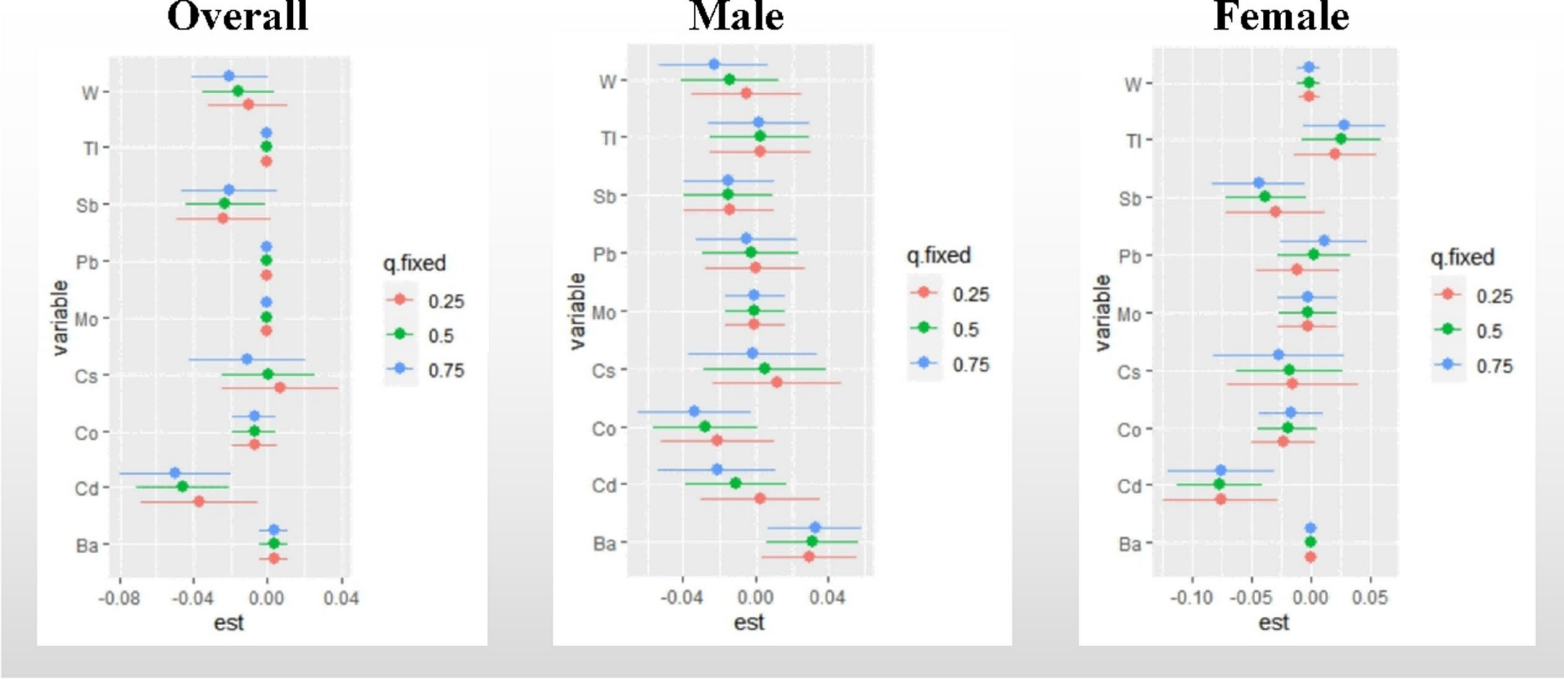
Effect of single metal changes on UFR at the 25, 50, and 75% quartiles for single metals (estimates and 95% confidence intervals). The model adjusted for sex, age, race, educational attainment, BMI, smoking status, cardiac history, systolic blood pressure, ln-urine creatinine, serum glucose, AST, and eGFR. in the gender-stratified analysis, confounders other than sex were included. “est” was defined as the association between a single metal element and UFR. 95% confidence intervals excluding any percentile estimate of 0 were considered statistically significant.

Single metal effect: Figure 5 presents a summary of the risk on UFR when a single metal was increased from the 25th to the 75th percentile, while other metals were fixed at different percentiles (25th, 50th, and 75th). Significant negative associations with UFR were observed for Cd (when other metals were fixed at the 25th, 50th, and 75th percentiles) in all study groups, except for the male group. In the overall participants, a statistically significant negative correlation was found between Sb and UFR (when other metals were fixed at the 50th percentile). The posterior inclusion probability (PIP) analysis indicated that Cd (PIP = 1) and Cs (PIP = 0.9136) contributed the most to the effect on UFR, followed by Sb (PIP = 0.1996). Among males, Ba showed a significant positive correlation with UFR, while Co exhibited a statistically significant negative correlation when other metals were fixed at the 75th percentile. Among females, a statistically significant negative correlation was observed between Sb and UFR when other metals were fixed at the 50th and 75th percentiles. The PIP analysis revealed that Cd (PIP = 0.9994) and Cs (PIP = 0.9663) had the highest contributions to the effect on UFR. Detailed PIP results can be found in Supplementary Table S2.

### 3.6. Sensitivity analysis

The results of the monometallic and polymetallic weighted linear regression analyses can be seen in Supplementary Figures S2, S3, respectively. After adjusting for all covariates, the monometallic model showed that urine Ba was significantly positively correlated with UFR in the overall participants (β = 0.023, 95% CI: 0.006, 0.041). Conversely, Cd (β = −0.034, 95% CI: −0.056, −0.013), Sb (β = −0.039, 95% CI: −0.062, −0.015), and W (β = −0.019, 95% CI: −0.033, −0.005) were significantly and negatively correlated with UFR. Females exhibited greater sensitivity to the response of metals in urine compared to males, as indicated by the significant negative associations between Cd (β = −0.057, 95% CI: −0.088, −0.027) and Sb (β = −0.082, 95% CI: −0.112, −0.052) with UFR in the female monometallic model.

Similar to the monometallic model, the polymetallic linear regression model, which includes all metals in the regression simultaneously, yielded comparable results. In males, UFR showed no significant associations with any of the metals, while in females, Ba (β = 0.029, 95% CI: 0.005, 0.053) was significantly and positively correlated with UFR, while Cd (β = −0.053, 95% CI: −0.084, −0.023) and Sb (β = −0.078, 95% CI: −0.111, −0.045) were significantly negatively correlated with UFR. Additionally, all variance inflation factors (VIF) in the polymetallic linear regression model were below 10. A summary of the different models is shown in Table 3.

**Table 3:** Summary results from different models.

	Overall	Male	Female
BKMR	Cd (−), Sb (−)	Ba (+), Co (−)	Cd (−), Sb (−)
WQS negative maximum weight	Sb (−), Cd (−), Cs (−), W (−)	Sb (−), Cs (−), Pb (−), Cd (−)	Cd (−), Co (−), Sb (−)
WQS positive maximum weight	Ba (+), Tl (+), Pb (+)	Tl (+), Co (+), Ba (+), W (+)	Ba (+), Pb (+), W (+)
Qgcomp negative weight	Sb (−), Cd (−), Co (−), Cs (−), W (−), Pb (−)	Co (−), Sb (−), W (−), Cd (−), Cs (−)	Sb (−), Cd (−), Co (−), Cs (−), W (−), Pb (−)
Qgcomp positive weight	Tl (+), Ba (+), Mo (+)	Ba (+), Tl (+), Mo (+)	Tl (+), Ba (+), Mo (+)
Monometallic weighted linear regression analyses	Ba (+), Cd (−), Sb (−), W (−)	-	Cd (−), Sb (−)
Polymetallic weighted linear regression analyses	Ba (+), Cd (−), Sb (−), W (−)	–	Ba (+), Cd (−), Sb (−)

WQS, weighted quantile sum; qgcomp, quantile g-computation; BKMR: Bayesian kernel machine regression. * (+) means positive weight while (−) means negative weight.

## 4. Discussion

To our knowledge, no previous studies have investigated the association between exposure to a mixture of metals and mean UFR. In this study, we examined the relationship between urinary metal elements and average UFR in various populations using multiple mixture analysis models. Overall, the findings from our study were consistent across all the analyzed models. The combined results revealed a significant negative correlation between urinary metal mixture exposure and UFR, with notable gender differences, particularly in females who showed higher sensitivity. It is noteworthy that Cd and Sb appear to be the primary contributors to these results, with Cd exhibiting the highest negative weight. However, it is important to mention that while the BKMR model identified a significant positive correlation between Ba and UFR in males, as well as a significant negative correlation between Co and UFR, these findings were not consistently observed in other models.

Cd is a widely known toxic non-essential metal element that can cause significant effects on the body, even at low doses. It has a low excretion rate and an extended biological half-life, and long-term exposure can have harmful effects on the organs that store the metal. In the United States, the daily dietary intake of Cd primarily comes from cereals and bread (34%) and green leafy vegetables (20%) (30). Additionally, tobacco use is another major source of Cd exposure (31). Cd has been found to inhibit cellular antioxidant enzyme activity, promote lipid peroxidation, and induce oxidative stress responses (32, 33). Studies have demonstrated that even at low concentrations, Cd can bind to cell mitochondria, hindering oxidative phosphorylation and resulting in cell damage and apoptosis (34, 35). Furthermore, Cd exposure can adversely affect the human nervous system (36, 37). Cd can inhibit the release of acetylcholine, which may be a biological effect by interfering with calcium metabolism (38).

Animal studies have demonstrated that exposure to Cd for 3 months adversely affects the neurogenic and myogenic contractile activity of the rat detrusor (39). In a subacute toxicity study involving isolated rat detrusor muscle, Cd was found to decrease contractile activity mediated by electrical field stimulation, acetylcholine (ACh), and adenosine triphosphate (ATP) (10). ACh and ATP are the primary neurotransmitters involved in bladder smooth muscle contraction. The toxicological mechanisms and the results of animal experiments of Cd mentioned above align with our findings. We observed a significant negative linear correlation between Cd and UFR, even at low doses. However, this correlation was not significant in male subjects. This suggests that daily Cd exposure at low doses can also cause abnormal bladder function and affect the contractile activity of the detrusor muscle of the bladder.

Sb is a heavy element widely present in the environment and extensively used in modern industry. Daily life sources of Sb exposure include diet, atmospheric pollution, drugs, and occupational settings (40). High levels of antimony are commonly found in proximity to smelters (41), with waste incineration and fossil fuel combustion also contributing to its presence (42). In addition, the use of plastic products makes food more susceptible to Sb contamination (43). Studies have demonstrated that the inorganic form of Sb exhibits a strong affinity for thiol groups, leading to intracellular glutathione depletion. Furthermore, Sb impairs glutathione peroxidase activity, reducing free glutathione levels and increasing cellular vulnerability to oxidative stress (44). Even at low doses, Sb significantly impacts mitochondrial function, decreasing mitochondrial membrane potential, respiratory enzyme complex activity (I/II/III/IV), ATP/ADP ratio, and ATP concentration (45). Sb exposure also inhibited intracellular pyruvate dehydrogenase activity, causing an increase in anaerobic glycolysis and resulting in a decrease in intracellular ATP levels. These findings suggest that antimony can cause damage to mitochondria. Typically, the respiratory and cardiovascular systems are mainly affected after Sb exposure (40). Experimental studies have revealed that the administration of antimony potassium tartrate induces cardiac fiber degeneration and connective tissue damage, even at low doses (46). In addition, it has been reported that Sb may be neurotoxic and can lead to neuronal apoptosis (47).

To our knowledge, no studies investigating the relationship between Sb and bladder function have been reported. In our study, we observed that the impact of Sb on mean UFR resembled that of Cd. This similarity suggests that Sb may reduce bladder detrusor muscle contractility through mechanisms involving cellular oxidative stress, inhibition of oxidative phosphorylation, apoptosis promotion, and damage to the nervous system. However, further research is required to fully elucidate the exact underlying mechanism.

Significant gender differences were observed in the effects of metal mixtures, as revealed by our study. Multiple mixture analysis models demonstrated a negative correlation between mean UFR and urinary metal mixtures in both males and females, with females displaying greater sensitivity. This finding aligns with previous studies, including research on the differential neurodevelopmental impact of prenatal and/or postnatal exposure to mercury, lead, manganese, cadmium, and arsenic in children (11). Furthermore, gender disparities were identified in the association between blood and urine metal mixtures and cancer mortality, with females exhibiting higher susceptibility to metal exposure (2). Notably, studies have reported a significant relationship between plasma Cu and glycosylated hemoglobin (HbA1c) exclusively in females, while no such association was observed in males (48).

Several potential mechanisms may account for this phenomenon. Firstly, differences in hormone levels between males and females can contribute to variations in metal metabolism (49). Secondly, gender differences in redox homeostasis, characterized by glutathione metabolism, may play a role (50). Additionally, genetic polymorphisms and differences in gene expression between sexes determine the differences in sensitivity to metals (51). Lastly, disparities in diet and behavioral habits between men and women can also have an impact (52). Notably, women’s unique physiological mechanisms, such as their propensity to experience greater iron loss, can result in increased metal absorption and subsequent enrichment and accumulation in their bodies (53).

This study possesses several advantages. First, the data were obtained from a large cross-sectional survey research program organized by the CDC, ensuring high data credibility, a large sample size, and generalizability of the findings to the overall population. Second, a range of mixture analysis models, including WQS regression, qgcomp, weighted multiple linear regression, and BKMR models, were utilized to comprehensively assess the relationship between metal mixtures and mean UFR. Traditional linear regression is inadequate for evaluating the combined effects of metal mixtures since their combined effect cannot be simply calculated as the sum of individual effects (54). The WQS model is able to explore the effect of mixed exposure burden on the results in one direction at a time, but since the WQS model takes quantile calculations for exposures, this may lose some of the information about the exposures. Additionally, the WQS regression model needs to satisfy the directional homogeneity assumption and also assumes that individual exposures have linear and additive effects. In contrast, qgcomp allows simultaneous validation of the correlation between exposure and outcome from both directions and calculation of weights for each of the two directions, while allowing for nonlinearity and nonadditivity of the effects of individual exposures and whole mixtures (28). BKMR models are valuable statistical tools for exploring combined mixture effects, offering linear or nonlinear response functions and visualizations for improved identification of key contaminants. However, the BKMR model is limited in assessing the impact of co-exposure patterns of high- and low-level metals. Therefore, these models can complement each other and undergo cross-validation to assess mixture exposure, and the subsequent joint interpretation will also facilitate the determination of specific exposure risks.

This study has a number of limitations. Firstly, it is important to note that it is a cross-sectional study, which means that it only represents the participants’ state at the time of testing. Consequently, no causal inferences can be drawn from the analysis results, and further prospective studies are required to support the final conclusions. Secondly, the UFR data available in the NHANES database do not include peak UFR values or the systolic and diastolic values of the detrusor muscle, which directly reflect bladder contractile function. Nonetheless, the mean UFR data provided by NHANES can still serve as a valid reference indicator for assessing bladder function (16, 17, 55).

## 5. Conclusion

In conclusion, our study revealed significant negative correlations between urinary metal mixture exposure and mean UFR in US adults. Moreover, these associations exhibited notable gender specificity. Higher urinary levels of Cd and Sb were identified as potential key factors contributing to the decrease in mean UFR. These findings underscore the potential detrimental impact of environmental metal exposure on bladder function. Further prospective studies are warranted to elucidate the underlying mechanisms and confirm the observed gender difference.

## Data availability statement

The raw data supporting the conclusions of this article will be made available by the authors, without undue reservation.

## Ethics statement

NHANES study was conducted under the authorization of the National Center for Health Statistics (NCHS) Ethics Review Committee, and all participants provided informed consent. The patients/participants provided their written informed consent to participate in this study.

## Author contributions

SZ contributed to the conception and design of the study, and was responsible for the overall content as guarantor. SZ and HT were responsible for data collection and checking, performed the data analysis, interpretation, and manuscript drafting. MZ supervised the project administration. All authors contributed to the article and approved the submitted version.

## Conflict of interest

The authors declare that the research was conducted in the absence of any commercial or financial relationships that could be construed as a potential conflict of interest.

## Publisher’s note

All claims expressed in this article are solely those of the authors and do not necessarily represent those of their affiliated organizations, or those of the publisher, the editors and the reviewers. Any product that may be evaluated in this article, or claim that may be made by its manufacturer, is not guaranteed or endorsed by the publisher.
