# Supplementary material for: Sex-specific associations between nine metal mixtures in urine and urine flow rate in US adults: NHANES 2009–2018
Source: Front Public Health. 2023 Jul 28;11:1241971. doi: 10.3389/fpubh.2023.1241971 (PMC10420057; doi:10.3389/fpubh.2023.1241971)
Supplement: Supplementary file 1 [file Data_Sheet_1.pdf]

Supplementary Materials

Sex-specific associations between nine metal mixtures in urine and urine flow rate in US adults: NHANES 2009-2018

Table S1. The detection rate of each specific metal element

	2009-2010		2011-2012		2013-2014		2015-2016		2017-2018		
	At or above the detection limit	Below lower detection limit	At or above the detection limit	Below lower detection limit	At or above the detection limit	Below lower detection limit	At or above the detection limit	Below lower detection limit	At or above the detection limit	Below lower detection limit	Overall detection rate(%)
Ba	2836	12	2486	18	2657	7	3041	20	2797	11	99.5%
Cd	2544	304	1943	561	2181	483	2282	779	2208	600	80.4%
Co	2830	18	2488	16	2662	2	3058	3	2805	3	99.7%
Cs	2848	0	2503	0	2664	0	3060	1	2808	0	100%
Mo	2848	0	2504	0	2664	0	3060	0	2808	0	100%
Pb	2768	80	2404	100	2636	28	3049	12	2390	15	98.3%
Sb	2116	731	1523	981	2065	599	2530	531	2297	511	75.8%
Tl	2833	15	2490	14	2643	21	3053	8	2802	6	99.5%
W	2558	289	2154	337	2232	432	2744	316	2541	267	88.2%

Note:barium (Ba), cadmium (Cd), cobalt (Co), cesium (Cs), molybdenum (Mo), lead (Pb), antimony (Sb), thallium (Tl), and tungsten (W).

Table S2. Distribution of metallic elements in urine (N=7733), NHANES, USA, 2009-2018.

Characteristic	Overall	Sex Group		
	**Overall**, N = 7733 (100%)[1]	**female**, N = 3921 (51%)[1]	**male**, N = 3812 (49%)[1]	**P-value**[2]
**Ba (ug/L)**	1.17 (0.56, 2.25)	1.05 (0.50, 2.14)	1.29 (0.63, 2.45)	<0.001
**Cd (ug/L)**	0.19 (0.09, 0.36)	0.20 (0.09, 0.40)	0.18 (0.09, 0.33)	0.03
**Co (ug/L)**	0.36 (0.21, 0.59)	0.37 (0.21, 0.65)	0.36 (0.22, 0.55)	0.071
**Cs (ug/L)**	4.2 (2.6, 6.5)	3.8 (2.3, 6.3)	4.7 (3.0, 6.7)	<0.001
**Mo (ug/L)**	36 (18, 63)	31 (16, 58)	40 (21, 69)	<0.001
**Pb (ug/L)**	0.35 (0.19, 0.62)	0.30 (0.16, 0.53)	0.41 (0.23, 0.71)	<0.001
**Sb (ug/L)**	0.04 (0.03, 0.08)	0.04 (0.02, 0.07)	0.05 (0.03, 0.08)	<0.001
**Tl (ug/L)**	0.16 (0.09, 0.25)	0.14 (0.08, 0.24)	0.17 (0.10, 0.26)	<0.001
**W (ug/L)**	0.06 (0.03, 0.12)	0.05 (0.02, 0.10)	0.07 (0.03, 0.13)	<0.001

Note:[1]N (unweighted) (%); Median (IQR)

[2]chi-squared test with Rao & Scott's second-order correction; Wilcoxon rank-sum test for complex survey samples

barium (Ba), cadmium (Cd), cobalt (Co), cesium (Cs), molybdenum (Mo), lead (Pb), antimony (Sb), thallium (Tl), and tungsten (W).

Table S3. Detailed PIP results for the BKMR model for each study group

	Metals	Overall PIP	Male PIP	Female PIP
1	Ba	0.0001	0.0138	0
2	Cd	1	0.3293	0.9994
3	Co	0.0007	0.0702	0.02
4	Cs	0.9136	0.0562	0.9663
5	Mo	0	0.0006	0.0186
6	Pb	0	0.0136	0.0716
7	Sb	0.1996	0.0058	0.2808
8	Tl	0	0.0041	0.0031
9	W	0.0077	0.0639	0.004

Note:barium (Ba), cadmium (Cd), cobalt (Co), cesium (Cs), molybdenum (Mo), lead (Pb), antimony (Sb), thallium (Tl), and tungsten (W). PIP,posterior inclusion probability.The model adjusted for sex, age, race, educational attainment, BMI, smoking status, cardiac history, systolic blood pressure, ln-urine creatinine, serum glucose, AST, and eGFR. in the gender-stratified analysis, confounders other than sex were included.

Figure S1. Nonlinear exposure-response relationship between single metals and UFR in BKMR model.

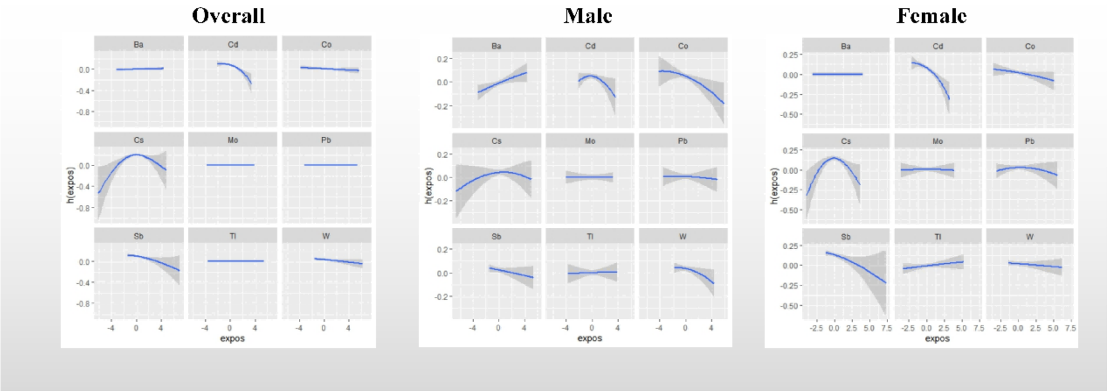

The model adjusted for sex, age, race, educational attainment, BMI, smoking status, cardiac history, systolic blood pressure, ln-urine creatinine, serum glucose, AST, and eGFR. in a sex-stratified analysis, confounders other than sex were included. h(expos) can be interpreted as the relationship between metallic substances and UFR.

Figure S2. Forest plot for Monometallic weighted linear regression analysis

Monometallic weighted linear regression

Subgroup	Beta (95%CI)	P value
**Overall**		
Ba	0.023(0.006,0.041)	0.011
Cd	-0.034(-0.056,-0.013)	0.003
Co	-0.004(-0.031,0.022)	0.742
Cs	0.009(-0.028,0.046)	0.639
Mo	-0.002(-0.024,0.02)	0.849
Pb	0.005(-0.019,0.028)	0.699
Sb	-0.039(-0.062,-0.015)	0.002
Tl	0.02(-0.013,0.052)	0.235
W	-0.019(-0.033,-0.005)	0.011
**Male**		
Ba	0.026(-0.002,0.054)	0.072
Cd	-0.003(-0.032,0.027)	0.86
Co	0.002(-0.039,0.043)	0.927
Cs	0.021(-0.033,0.074)	0.452
Mo	0(-0.036,0.035)	0.985
Pb	0.019(-0.013,0.051)	0.244
Sb	0.001(-0.029,0.031)	0.937
Tl	0.03(-0.013,0.074)	0.175
W	-0.018(-0.042,0.007)	0.164
**Female**		
Ba	0.022(-0.001,0.044)	0.063
Cd	-0.057(-0.088,-0.027)	<0.001
Co	-0.016(-0.051,0.019)	0.384
Cs	0.002(-0.047,0.051)	0.95
Mo	-0.002(-0.034,0.03)	0.919
Pb	-0.01(-0.043,0.023)	0.553
Sb	-0.082(-0.112,-0.052)	<0.001
Tl	0.01(-0.031,0.052)	0.623
W	-0.019(-0.04,0.003)	0.09

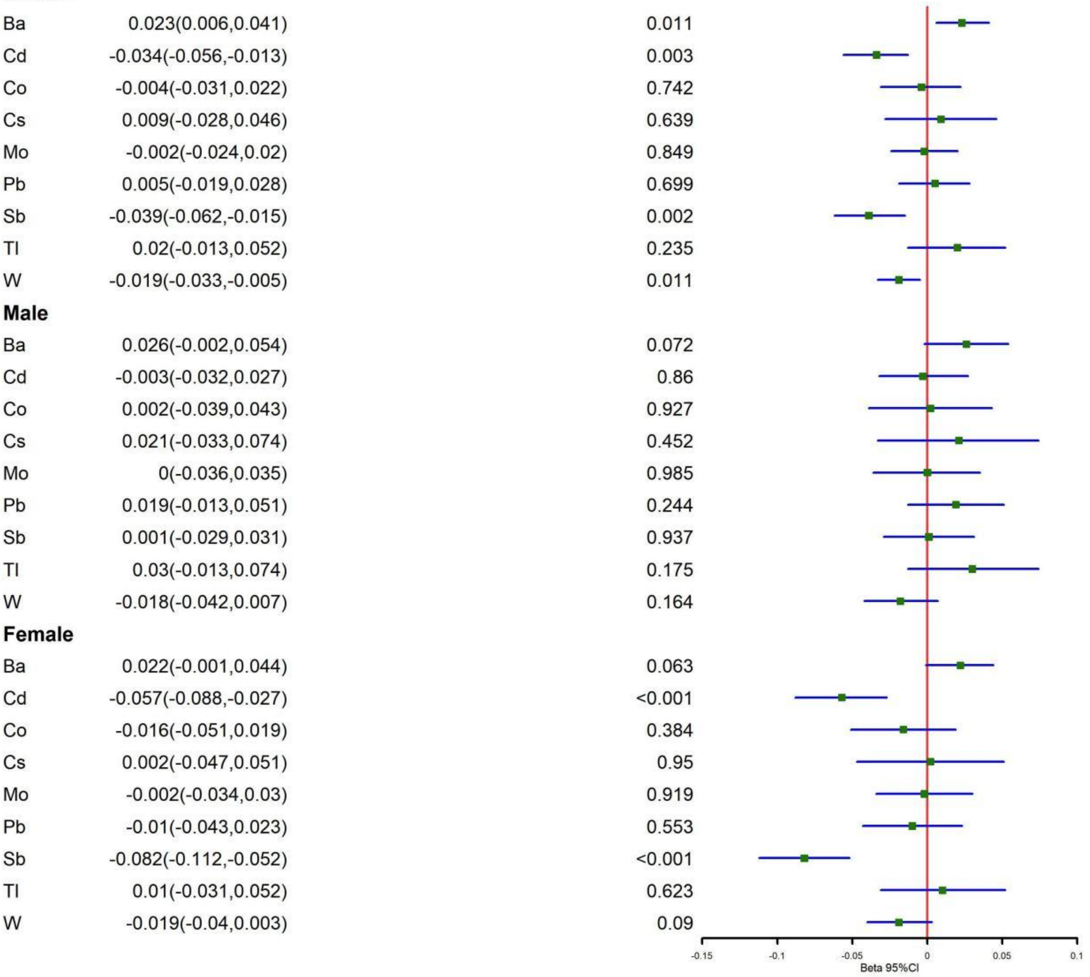

Note:barium (Ba), cadmium (Cd), cobalt (Co), cesium (Cs), molybdenum (Mo), lead (Pb), antimony (Sb), thallium (Tl), and tungsten (W). CI,Confidence interval. The model adjusted for sex, age, race, educational attainment, BMI, smoking status, cardiac history, systolic blood pressure, ln-urine creatinine, serum glucose, AST, and eGFR. in the gender-stratified analysis, confounders other than sex were included.

Figure S3. Forest plot for polymetallic weighted linear regression analyses.

Polymetallic weighted linear regression models

Subgroup	Beta (95%CI)	P value
**Overall**		
Ba	0.026(0.007,0.046)	0.012
Cd	-0.034(-0.056,-0.012)	0.004
Co	-0.013(-0.042,0.015)	0.356
Cs	-0.012(-0.053,0.029)	0.561
Mo	0.009(-0.016,0.033)	0.493
Pb	0.01(-0.014,0.035)	0.417
Sb	-0.036(-0.061,-0.011)	0.007
Tl	0.024(-0.01,0.058)	0.179
W	-0.019(-0.035,-0.003)	0.028
**Male**		
Ba	0.028(0,0.056)	0.059
Cd	-0.005(-0.033,0.023)	0.717
Co	-0.012(-0.056,0.032)	0.589
Cs	-0.003(-0.062,0.056)	0.914
Mo	0.008(-0.027,0.044)	0.643
Pb	0.013(-0.016,0.042)	0.379
Sb	0.002(-0.028,0.032)	0.902
Tl	0.028(-0.019,0.075)	0.252
W	-0.02(-0.044,0.004)	0.103
**Female**		
Ba	0.029(0.005,0.053)	0.022
Cd	-0.053(-0.084,-0.023)	0.001
Co	-0.023(-0.058,0.012)	0.208
Cs	-0.012(-0.07,0.045)	0.679
Mo	0.009(-0.027,0.046)	0.612
Pb	0.007(-0.027,0.041)	0.67
Sb	-0.078(-0.111,-0.045)	<0.001
Tl	0.021(-0.027,0.068)	0.397
W	-0.014(-0.038,0.011)	0.279

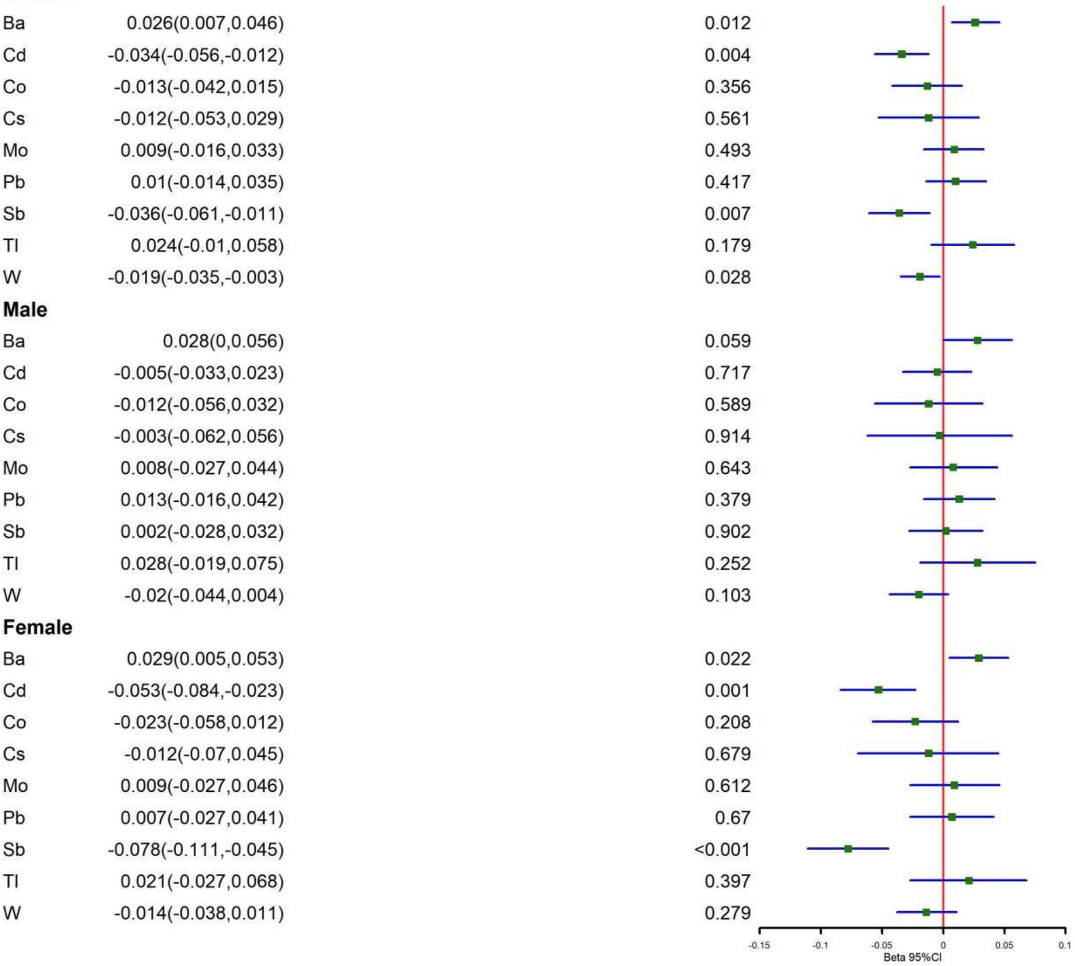

Note:barium (Ba), cadmium (Cd), cobalt (Co), cesium (Cs), molybdenum (Mo), lead (Pb), antimony (Sb), thallium (Tl), and tungsten (W). CI,Confidence interval. The model adjusted for sex, age, race, educational attainment, BMI, smoking status, cardiac history, systolic blood pressure, ln-urine creatinine, serum glucose, AST, and eGFR. in the gender-stratified analysis, confounders other than sex were included.
